# On the Application of Quantitative EEG for Characterizing Autistic Brain: A Systematic Review

**DOI:** 10.3389/fnhum.2013.00442

**Published:** 2013-08-05

**Authors:** Lucia Billeci, Federico Sicca, Koushik Maharatna, Fabio Apicella, Antonio Narzisi, Giulia Campatelli, Sara Calderoni, Giovanni Pioggia, Filippo Muratori

**Affiliations:** ^1^Institute of Clinical Physiology, National Council of Research (CNR), Pisa, Italy; ^2^IRCCS Stella Maris Foundation, Pisa, Italy; ^3^Electronics and Computer Science, Faculty of Physical and Applied Sciences, University of Southampton, Southampton, United Kingdom; ^4^Department of Developmental Medicine, University of Pisa, Pisa, Italy

**Keywords:** autism-spectrum disorder, quantitative electroencephalography, coherence, asymmetry, non-linear techniques

## Abstract

Autism-Spectrum Disorders (ASD) are thought to be associated with abnormalities in neural connectivity at both the global and local levels. Quantitative electroencephalography (QEEG) is a non-invasive technique that allows a highly precise measurement of brain function and connectivity. This review encompasses the key findings of QEEG application in subjects with ASD, in order to assess the relevance of this approach in characterizing brain function and clustering phenotypes. QEEG studies evaluating both the spontaneous brain activity and brain signals under controlled experimental stimuli were examined. Despite conflicting results, literature analysis suggests that QEEG features are sensitive to modification in neuronal regulation dysfunction which characterize autistic brain. QEEG may therefore help in detecting regions of altered brain function and connectivity abnormalities, in linking behavior with brain activity, and subgrouping affected individuals within the wide heterogeneity of ASD. The use of advanced techniques for the increase of the specificity and of spatial localization could allow finding distinctive patterns of QEEG abnormalities in ASD subjects, paving the way for the development of tailored intervention strategies.

## Introduction

Autism-Spectrum Disorders (ASD) are neurodevelopmental conditions characterized by deficits in social communication and interactions and by the presence of repetitive patterns of behavior, interests, and activities (American Psychiatric Association, 2013). These features appear in early childhood, tend to persist life-long, and often lead to poor outcome in adulthood. Recent epidemiological studies estimate the prevalence of ASD to be 1 in 88 children in the USA (Centers for Disease Control and Prevention, 2012). Despite an extensive research, there is still much debate about the morphological, functional, and neuropsychological characteristics of the “autistic” brain (Schipul et al., 2011; Calderoni et al., 2012; Muratori et al., 2012; Narzisi et al., 2012), and thus the neural basis of altered behaviors in ASD remains largely unclear. Several neuroimaging and neurophysiological techniques have been used in order to understand the correlation between brain functionality and autistic behavior. Among them, Quantitative Electroencephalography (QEEG) is currently receiving great interest and it is increasingly used in studies on neurodevelopmental disorders, especially the ASD. It has been found relevant for evaluating heterogeneity of behavioral disorders, treatment responses, and outcomes amongst other issues (Sheikhani et al., 2009). The ease and simplicity of the EEG procedure and its millisecond resolution of brain activity coupled with standardized analysis protocols provides an opportunity for elaborate analysis of brain functions and dysfunctions. Emerging EEG analysis techniques that involve interesting applications of signal analysis protocols have given us new and exciting measures of brain function.

According to the American Academy of Neurology, QEEG is defined as “… The mathematical processing of digitally recorded EEG in order to highlight specific waveform components, transform the EEG into a format or domain that elucidates relevant information, or associate numerical results …” (Nuwer, 1997). Therefore, QEEG applies computerized mathematical algorithms to transform raw EEG data into a number of frequency bands of interest. Five wide frequency bands are usually studied, typically defined as delta (1.5–3.5 Hz), theta (3.5–7.5 Hz), alpha (7.5–12.5 Hz), beta (12.5–30 Hz), and gamma (30–70 Hz) (Steriade et al., 1990). In addition, although not included in the standard classification of EEG bands, a further alpha-like rhythm, called mu rhythm, has been extensively studied in the research on ASD (Oberman et al., 2005, 2008; Barnier et al., 2007), since mu suppression during the observation of biological actions is thought to reflect mirror neuron system (MNS) functioning and, in its turn, MNS dysfunction has been proposed to explain the core social deficits observed in ASD (Williams et al., 2001). EEG recordings may be performed at rest, in both closed and opened eye conditions, or while subjects perform specific tasks. Normative or control data are usually needed, in order to give meaning to the functional information obtained.

In this article we carried out a systematic survey of existing research to present an integrated view on how QEEG may help in characterizing autistic brain and present the future direction in the same domain. The paper is organized as follows: after briefly describing the fundamentals of QEEG in Section “QEEG Methods,” we reviewed the recent works on application of QEEG in characterizing the autistic brain in Section “QEEG in Autism.” Three particular directions have been surveyed in detail – QEEG during rest, during specific tasks, and how QEEG may be applied for classifying autistic subgroups. The paper is concluded and future research directions are discussed in Section “Discussion and Conclusion.”

## QEEG Methods

For QEEG analysis raw EEG data are collected non-invasively via a set of electrodes typically following an international 10/10 or 10/20 electrode placement configuration on the scalp. The collected data is then transformed into frequency domain using computerized algorithms (i.e., Fourier Transform, Welch Method) and scalp map of different frequency bands is obtained (Dumermuth and Molinari, 1987).

Absolute and relative spectral power (SP) consists in transforming the EEG traces from time domain into frequency domain providing information about the harmonic content of the signal. The spatial analysis provides information about the distribution of electrical activity in the brain and the interconnectivity among cortical regions measured through coherence and symmetry analyses.

EEG power spectrums are regulated by anatomically complex homeostatic systems in the various frequency bands. Brainstem, thalamic, and cortical processes involving large neuronal populations mediate this regulation, using all the major neurotransmitters. The spectrum is quite stable in healthy individuals because it is regulated by homeostatic regulation of neurotransmitters and can be abnormal in some psychiatric disorders due to the dysfunction of this regulation (Hughes and John, 1999).

Coherence measures the degree of coupling between signals generated by specific neuronal assemblies, which are located in proximity of the recorded electrodes. Coherently oscillating neuronal assemblies exhibit electrical activity with common spectral properties. When a coherent oscillation occurs these neural groups can effectively communicate, because their communication windows for input and for output are open at the same times (Fries, 2005). The coherence pattern is flexible, changing according during specific cognitive or motor task and allowing the maintenance of our cognitive flexibility (Fries, 2005).

Brain asymmetry is due to hemispheric specialization, so that the global neural activity is not the same in the two hemispheres. However it has been shown that hemispheric differences in competence are not fixed and structure-dependent but are subject by dynamic processes. According to this view asymmetry can vary inter- and intra-individually according to arousal or other factors (Hugdahl, 1996).

Taken together, frequency and spatial information, and their modification over consecutive EEG epochs, provides a quantitative view of the dynamic evolution of connectivity between different brain areas, getting therefore cues on the functional organization of underlying neuronal networks in static and dynamic settings.

Before applying a quantitative analysis, a pre-processing step needs to be performed. First of all the signal is segmented in epochs of the same length and visually inspected, in order to reject those epochs with evident artifacts. Any remaining artifact is then removed by using high-pass, low-pass, and notch filters.

In most cases, frequencies below 0.5 Hz, due to movement artifacts, and higher than 60 Hz, afflicted by muscle artifacts, are filtered out, although this latter operation impairs the possibility to analyze gamma band. Notch filter allows removing artifacts caused by electrical power lines (50 or 60 Hz according to the country).

After preprocessing, spectral analysis can be applied to the signal. The Power Spectral Density (PSD) can be calculated by transforming the time domain signal to the frequency domain, using different techniques such as the Fast Fourier Transform (FFT) or the Welch method (Welch, 1967). From PSD the absolute power of the signal can be computed. However, since the absolute power measures may vary significantly in humans, it is more useful to calculate the power ratios among bands, which show less variability among subjects and are less affected by artifacts. Power ratios are expressed as a percentage, and are obtained by dividing the absolute power of a specific band by the total absolute power of the spectrum. Power ratio can be calculated also between only two bands (e.g., α/θ) or between band sets (e.g., α + β/θ + δ).

Classical spectral analysis techniques, like the FFT, are very useful when analyzing stationary signals. Nevertheless, when dealing with non-stationary signals, as is the case of EEG, they show the big disadvantage of not preserving information on the temporal evolution/localization of the frequency components. This occurs because changes in frequency content, at a given time instant, cause changes to all the Fourier coefficients and therefore it is not possible to localize at which times these frequencies occur. In QEEG analysis, temporal information is important to detect and monitor changes in brain activity at different time-scales following a specific event. For this reason in some studies other techniques as the Short-Time Fourier Transform (STFT) have been used (Sheikhani et al., 2007). STFT can be interpreted as the Fourier transform of the signal observed through a sliding time window of finite duration. The STFT allows constructing the signal spectrogram, which is an image representation of the magnitude of Fourier coefficients within that time window and therefore describes the frequency contents of the signal in the neighborhood (bounded by the time window) of the selected time instant (Walter, 1963).

Spectral analysis is often associated with spatial analysis that allows characterizing relationships between activities of different brain areas. The spatial information may be mainly derived from QEEG data through symmetry and coherence analysis. The symmetry between the two hemispheres can be computed using the Brain Symmetry Index (BSI) (John et al., 1977; Van Putten et al., 2004). The BSI captures a particular asymmetry in SP between hemispheres, and is normalized between 0 (perfect symmetry) and 1 (maximal asymmetry). Asymmetry is defined by the difference on the EEG absolute power between homologous contralateral electrodes and it is calculated as:

$$\text{BSI}=\left(\text{LH}-\text{RH}\right)∕\left(\text{LH}+\text{RH}\right)$$

where LH is the absolute power at one electrode in the left hemisphere and RH at its homologous electrode in the right hemisphere. On the other hand, the coherence function is a measure of the degree of association or coupling of frequency spectra between two EEG signals simultaneously recorded from different scalp locations per frequency band. Mathematically coherence is defined as the squared cross-correlation between two waveforms within a specific frequency band that has been normalized for amplitude (Otnes and Enochson, 1972). It is assumed to be an index of functional coupling between different brain areas. In neuroscience the coherence measure is generally distinct from synchrony, which refers to signals oscillating at the same frequency with identical phases (Singer, 1999).

Finally, another interesting QEEG index, recently introduced by Pop-Jordanova and Pop-Jordanov (2005), is the “brain rate.” This is calculated as the EEG spectrum weighted frequency with the following formula:

$$f_{b}\sum_{i}P_{i}=\sum_{i}f_{i}V_{i}∕V\;\text{with}\;V=\sum_{i}V_{i}$$

where the index corresponds to the frequency band (*i* = 1 for delta, *i* = 2, for theta, etc.), *f_i_* is the mean frequency of the corresponding band and *V_i_* is the mean amplitude of the electric potential associated to each band. The brain rate can thus be defined as an integral state attribute correlated to brain electric, mental, and metabolic activity (Pop-Jordanova and Pop-Jordanov, 2005).

The techniques described above are all linear methods and are the most commonly applied in the analysis of QEEG data. Given the non-linear nature of EEG, however, non-linear methods could be more suitable for the analysis of this signal. Although less conventional, a set of non-linear techniques have been sometimes used in QEEG analysis, allowing to obtain new information not detectable through linear methods, such as non-linear interactions and the complexity and stability of underlying brain sites. Some of these techniques, like higher-order statistical analysis, complexity analysis, and the phase synchronization analysis, have been applied to the study of QEEG signals in ASD.

Finally, the analysis of microstates represents another promising technique although not yet used in ASD research.

Among the higher-order statistical analysis techniques, the bispectral analysis is an advanced technique that quantifies quadratic non-linearities amongst the components of the EEG signal. In particular it measures the phase relationships between different frequency components and on that basis quantifies the degree of dependence amongst these components. Bispectrum is computed by the Fourier transform of the third order cumulant (a statistical measure of correlation). As the bi-spectrum depends not only on phase coupling but also on the power, it can be normalized in order to make it sensitive only to changes in phase coupling. This normalized bispectrum is then termed as bicoherence (for a detailed description of the mathematical bases of the bispectral analysis, see Sigl and Chamoun, 1994).

Another interesting property of EEG signal is complexity, which reflects random fluctuations over multiple time scales in the dynamics of neural networks, thus providing insights about neural connectivity. The most interesting methods employed for computing complexity of EEG signals are entropy and fractality.

Entropy is a physical measure related to the amount of disorder in a system, and it describes the irregularity or unpredictability characteristics of a signal.

Since regularity is not necessarily correlated with complexity, the quantification of complexity of EEG signals can be computed using the multiscale entropy (MSE), which measures the entropy across multiple time scales (Costa et al., 2002). This method is based on the principle that biological systems are modulated by multiple mechanisms, which interact over multiple temporal scales generating complex data. Another quantity that identifies the degree of complexity of a system is the fractal dimension. This is a non-integer number describing the self-similarity of a system: the whole can be fitted by parts of it by shifting and stretching (Mandelbrot, 1977).

The phase synchrony analysis may be useful when needed to analyze the phase relationships between EEG signals at different electrodes, independently of their amplitude (Lachaux et al., 1999). The basic idea of this technique is to generate an analytic signal from which a phase, and a phase difference between two signals, can be defined. On the basis of this phase difference a phase synchronization index can be computed, which will be zero if the signals under investigation are not synchronized and will be one for a constant phase difference.

Finally, the technique of functional microstates allows studying brief transactions occurring in the brain in the time range of milliseconds. Microstates are defined as time periods, of 80–120 ms, during which the potential distribution over the scalp shows stable topographical configuration after which a rapid transition to another stable configuration (another microstate) occurs (Lehmann et al., 1987). Microstates could be considered as the basic blocks of human information processing (Lehmann, 1990), reflecting the interactions between environmental information and the subject’s previous knowledge and internal state. Microstates can only repeat several times within a period so that a cluster approach can be adopted to identify different classes of electrical states composing the EEG signal. Several statistical measures can then be extracted and related to the different experimental conditions and microstate class, such as mean microstate duration, mean number of microstates per second, or the percentage time covered by each state (Koenig et al., 2002) (for a detailed mathematical description of the non-linear analysis techniques described above, see Tong and Thakor, 2009).

Quantitative electroencephalography data are usually obtained using commercial or free software that are able to extract the most common features of EEG signals. The use of advanced techniques such as Independent Component Analysis (ICA), and neuroimaging techniques such as Low Resolution Electromagnetic Tomography (LORETA) (Pascual-Marqui et al., 1994) are used to map the actual sources of the cortical rhythms. These advanced techniques may, therefore, represent a promising approach to understand the dynamics and functions of the brain in a number of neurological diseases, including ASD.

## QEEG in Autism

Quantitative electroencephalography has been adopted in several studies for the assessment of ASD with the aim of finding out quantitative indices characterizing brain functions. An understanding of how this evolving technique can aid future research in ASD is very important.

Several studies highlight QEEG capacity to classify subjects with ASD from controls, or different subgroups of ASD. Moreover, QEEG has been also applied as a tool for therapeutic intervention through a neurofeedback approach, although its use in ASD is still poorly reported in literature. A description of neurofeedback methods and of its application in ASD, however, is beyond the aim of this article, and readers may refer to dedicated original researches and reviews (Pineda et al., 2008; Kouijzer et al., 2009, 2010; Coben et al., 2010).

In the following subsections, we will review the main applications of QEEG in three different scenarios: (1) closed or open-eyes rest condition; (2) subjects performing specific tasks; (3) subtyping of ASD through QEEG information. All the studies reviewed here are summarized in Table 1. This research provides evidence for the utility of QEEG in extracting objective measures that can characterize brain activity in different conditions.

**Table 1:** **Summary of QEEG studies in autism**.

Study	Design	Participants	Measures	Results in ASD
**QEEG DURING REST**				
Cantor et al. (1986)	10/20 System, open eyes	*n* = 11 ASD (age: 4–12 years, IQ = 37.45 ± 11.4)	PSD, coherence, symmetry	Higher percent delta and less alpha; higher coherence between and within, hemispheres; less asymmetry
		*n* = 88 TD (age: 5–15 years, IQ = 113.35 ± 9.5)	
		*n* = 18 Intellectually disabled (age: 5–15 years, IQ = 71.1 ± 12.6)	
		*n* = 13 TD toddlers (age: 16 months to 5 years, IQ = 121.0 ± 25.4)	
Chan et al. (2007)	10/20 System, open eyes	*n* = 66 ASD (age: 5–18 years, TONI = 83.36 ± 21.61)	PSD	Higher absolute delta and lower relative alpha; same results for all channel
		*n* = 90 TD (age: 6–12 years, TONI = 111.42 ± 16.16)	
Stroganova et al. (2007)	32 or 24 Electrode system, open eyes	*n* = 40 ASD (age: 3–8 years, IQ = 111 ± 8.29)	PSD, asymmetry	Higher prefrontal delta; leftward asymmetry at the temporal regions; symmetric mu rhythm in ASD across central sites
		*n* = 40 TD (age: 3–8 years)	
Coben et al. (2008)	10/20 System, closed eyes	*n* = 40 ASD (age: 6–11 years, IQ = 93 ± 16.8) *n* = 40 TD (age: 6–11 years, IQ = 98 ± 15.4)	PSD, coherence	Less absolute (left frontal and posterior region) and relative delta (left frontal and vertex regions) and higher absolute beta (midline regions) and relative theta (right posterior regions); less delta and theta intrahemispheric coherence; less inter-hemispheric coherences in delta and theta in frontal regions in delta, theta, and alpha in temporal regions and in delta, theta, and beta bands central/parietal/occipital regions
Murias et al. (2007)	128 Channels, closed eyes	*n* = 18 ASD (age: 18–38 years, IQ = 107.33 ± 13.96) *n* = 18 TD (age: 18–38 years, IQ = 106.11 ± 13.56)	PSD, coherence	Higher relative theta in primarily frontal and prefrontal regions, less relative alpha in primarily frontal/prefrontal and occipital/parietal regions and higher relative beta in occipital/parietal regions; higher coherence in theta and less coherence in alpha
Pop-Jordanova et al. (2010)	10/20 System, open eyes and closed eyes	*n* = 9 ASD (age: 3–6 years) database of TD	PSD, brain rate	Higher delta/theta; higher beta in open eyes than in closed eyes; reduction of brain rate in all regions
Mathewson et al. (2012)	128 Channels, open eyes and closed eyes	*n* = 15 ASD (age: 19–52 years, IQ = 100.9 ± 18.6) *n* = 18 TD (age: 18–38 years, IQ = 107.1 ± 11.9)	PSD, coherence, correlation with AQ	Higher alpha in eye opens; less alpha suppression in 01; no difference in coherence; negative correlation between alpha and preferential attention to detail in posterior and frontal regions both in eye open and eye closed; negative correlation between attention to details and coherence in eye opened in the right centro-parietal region and in eye closed in the parieto-occipital regions; negative correlation between alpha coherence in eye-open and social functioning in the right fronto-central region; positive correlation between theta coherence in the left centro-parietal region in eye-closed and social functioning
Sheikhani et al. (2007)	10/20 System, closed eyes	*n* = 10 ASD (age: 9.3 ± 1.8 years) *n* = 7 TD (age: 9.2 ± 0.7 years)	PSD (STFT and STFT-BW) and bispectrum	No differences in STFT or bispectrum; significant differences in STFT-BW over Fp1, F3, F7, T3, T5, and O1
**QEEG DURING REST**				
Ahmadlou et al. (2010)	10/20 System, closed eyes	*n* = 9 ASD (age: 6–13 years) *n* = 6 TD (age: 7–13 years)	Fractal dimension (HFD and KFD)	Significant difference in HFD in gamma, beta, and alpha and in KFD in gamma, beta, and delta
Thatcher et al. (2009)	10/20 System, open eyes	*n* = 54 ASD (age: 2.6–10.74 years) *n* = 241 TD (age: 2.2–11 years)	Phase synchronization analysis	Shorter phase shift durations in ASD in the alpha-1 frequency band (8–10 Hz); longer phase lock durations in ASD in the alpha-2 frequency band (10–12 Hz) and; differences in short and long inter-electrode pairs
Bosl et al. (2011)	64 Channels, open eyes	*n* = 46 HRA (age: 6–24 months) *n* = 33 TD (age: 6–24 months)	Multiscale Entropy (MSE) analysis	Reduced MSE in HRA subjects especially in 9–24 months range; discrimination between HRA and controls at 9 months with 80% of accuracy
Duffy and Als (2012)	32 Channels, open eyes	*n* = 463 ASD (age: 1–18 years) *n* = 571 TD (age: 1–18 years)	Coherence	High classification success between ASD and TD groups. decrease in short–distance coherence and increase in long-distance coherence in ASD group within a wide spectral range
**QEEG DURING SPECIFIC TASKS**				
Oberman et al. (2005)	10/20 System, tasks: (1) moving their own hand, (2) watching a video of a moving hand, (3) watching a video of two bouncing balls (non-biological motion), and (4) watching visual white noise	*n* = 10 ASD (age: 9–14 years, IQ > 80) *n* = 10 TD (age: 9–14 years)	PSD mu	Decreased mu only during the self-initiated hand movement
Orekhova et al. (2007)	10/20 System, sustained visual attention	*n* = 40 ASD (age: 3–8 years) *n* = 40 TD (age: 3–8 years)	PSD in high frequency bands	Higher power especially in gamma1 in midline, central, and parietal regions
Sheikhani et al. (2009)	10/20 System, tasks: (1) eye-closed condition, (2) eye-opened condition, (3–5) looking at three samples of Kanizsa shapes, (6) looking at mother’s picture upright and (7) inverted, (8) looking at stranger’s picture upright, and (9) inverted in frequency bands	*n* = 15 ASD (age: 6–11 years, IQ > 85) *n* = 11 TD (age: 6–11 years, IQ > 85)	PSD, spectrogram	Lower spectrogram criteria at Fp1, Fp2, and T6 in gamma and higher spectral power at FP1 and FP2 in open-eyes condition; difference in alpha at T3, F7, and C3 in looking at the inverted mother’s picture; difference in alpha and beta at F7, F4, F8, C4, Pz. In looking at a stranger’s picture inverted
Sheikhani et al. (2012)	128 Channels (reduced to 10/20), sustained visual attention	*n* = 17 ASD (age: 6–11 years, IQ > 85) *n* = 11 (age: 6–11 years, IQ > 85)	Spectrogram criteria	Lower spectrogram criteria in alpha, beta, and gamma especially in temporal and frontal regions in left hemisphere
Chan et al. (2011a)	Object recognition (OR) task	*n* = 21 ASD (age: 5–14 years, TONI = 101.86 ± 16.09) *n* = 21 TD (age: 5–14 years, TONI = 106 ± 14.59)	Coherence in theta	Elevated fronto-posterior coherences in left hemisphere; higher coherence in the left than in the right hemisphere; negative correlations between memory performance and the inter-hemispheric coherence
**QEEG DURING SPECIFIC TASKS**				
Chan et al. (2011b)	10/20 System, Go/No-Go task	*n* = 20 ASD (age: 7–14 years) *n* = 20 TD (age: 7–14 years)	PSD in theta source localization with LORETA	In the “Go” condition theta decreased in the anterior cingulate cortex (ACC), in the “No-Go” condition in the ACC and in the precuneus
Lushchekina et al. (2012)	10/20 System, tasks: (1) eye-closed condition, (2) counting during eye closed	*n* = 27 ASD (age: 5–7 years) *n* = 19 TD (age: 5–7 years)	PSD, coherence	Higher gamma in baseline condition; right-sided predominance of spectral power in alpha both at rest and during counting
Catarino et al. (2011)	10/20 System, detection tasks: (1) faces, (2) chairs	*n* = 15 ASD (age: 23.79–42.34 years, IQ = 119 ± 13)	MSE, PSD	Reduced MSE in ASD especially in parietal regions; higher MSE in response to faces in both groups; no differences in PSD
		*n* = 15 TD (age: 21.50–37.77 years, mean IQ = 119 ± 14)	
**QEEG FOR THE IDENTIFICATION OF AUTISTIC SUBGROUPS**				
Dawson et al. (1995)	10/20 System, open eyes	*n* = 28 ASD (age: 5–19 years, IQ = 119 ± 13)	PSD	Reduced delta and theta in the passive group in all brain regions and reduced alpha in the frontal regions
		*n* = 28 Chronological-age-matched TD (age: 5–19 years)	
		*n* = 24 Language-age-matched TD (age: 2–7 years)	
Sutton et al. (2005)	10/20 System, open eyes and closed eyes	*n* = 23 ASD (age: 9–14 years, IQ: 110.13 ± 21.21) *n* = 20 TD (age: 9–14 years IQ: 116.80 ± 11.69)	PSD	Higher alpha in anterior, central, and posterior cortical regions; more left-sided mid-frontal and central regions; subgroups with greater left-sided mid-frontal activity had greater social anxiety, greater general anxiety, greater social stress, and less satisfaction with interpersonal relations

*ASD, autism-spectrum disorder; TD, typical developing; HRA, “high risk” of autism*.

### QEEG during rest

Quantitative electroencephalography during rest conditions represents one of the most used applications of this technique in the field of autism research. Neural oscillations reflect the synchronous firing of large populations of neurons mediated by excitatory/inhibitory interactions and can be registered on the scalp. QEEG at rest may therefore inform, *in vivo*, on the balance between excitatory and inhibitory activities, which in turn could affect the cognitive and social functioning in ASD (Rubenstein and Merzenich, 2003; Bourgeron, 2009). The observation of spontaneous brain activity also allows characterizing different patterns in functional connectivity that might specify functionally relevant brain networks.

Finally, by studying power fluctuations of different bands it is possible to notice deviations from normal patterns that reflect the organization of the underlying system.

One of the first studies on QEEG in autism (Cantor et al., 1986) tried to examine if QEEG analysis could be used to differentiate low-functioning children with ASD from subjects with mental retardation without ASD, age-matched subjects with typical development and toddlers with typical development. Data were acquired during open-eyes rest condition and spectral as well as spatial analysis on the acquired data were performed.

Differences in PSD, coherence, and symmetry were found. In particular, children with ASD showed a significantly greater percentage delta and less alpha activity, higher degree of coherence between and within hemispheres, especially in delta and alpha band, and less amplitude asymmetry in every band. Interestingly, while ASD subjects significantly differentiate from age-matched controls and mentally retarded subjects, the pattern of activation was similar to the one obtained in typical toddlers, suggesting a maturational lag in cerebral functioning of subjects with ASD.

Chan et al. (2007) also studied PSD in children with ASD and in neurotypical controls by recording EEG in an open-eyes condition. In this study both low and high-functioning ASD children were included in the sample set. In consistence with the previous study, the ASD group showed significantly higher absolute delta and lower relative alpha. The authors attempted to localize the abnormalities in EEG signal, and found similar results at each channel suggesting that QEEG characteristics were not regionally specific, but were observed across all the cortex of children with ASD. This conclusion is also consistent with neuroimaging data indicating widespread brain abnormalities in ASD that include increased total intracranial volume (Courchesne et al., 2007), abnormal gray matter (Waiter et al., 2004; McAlonan et al., 2005), altered white matter (Barnea-Goraly et al., 2004; Billeci et al., 2012), and disrupted anatomical functioning (Belmonte and Yurgelun-Todd, 2003; Hubl et al., 2003).

Abnormalities in symmetry in ASD children were also found in the study of Stroganova et al. (2007). They acquired EEGs in open-eyes condition, in a large group of high-functioning children with ASD and in age-matched controls. PSD was calculated for delta, theta, and alpha frequency bands and BSI was computed. Atypical EEG asymmetry in children with ASD was found. In particular: (1) a broad-band leftward EEG asymmetry at the temporal and some adjacent regions that was absent in controls and (2) a symmetrically distributed mu rhythm in ASD across central sites of the left and right hemisphere, whereas in controls it was lateralized to the left. The first result could reflect structural asymmetries in the brain, although research on this issue is still inconclusive. For example, a decrease of deep white matter predominantly in the right hemisphere of ASD individuals has been highlighted in two recent studies (Boddaert et al., 2004; Waiter et al., 2005). However, further research is needed to adequately correlate structural and neurophysiologic data in ASD. Concerning the second result, the asymmetric distribution of mu is known to be linked to motor function and could mean that there is a greater down regulation of sensorimotor areas of the left hemisphere, involved in the control of the dominant right hand. On the contrary, the symmetrical distribution may be linked to a decreased control of motor function of the right hand.

Coben et al. (2008) demonstrated how the closed-eyes condition might cause changes in PSD and in coherence. In this study EEG was acquired on high-functioning children with ASD and controls and absolute and relative PSD for each band were computed. Moreover, intra-hemispheric and inter-hemispheric coherences were calculated. Opposite results in delta and beta band spectra with respect to the open-eyes condition were detected: in fact, a reduction in absolute and relative delta and an increase in absolute beta and relative theta distinguished ASD subjects from controls.

Also the coherence analysis revealed opposite results with respect to the open-eyes condition. In this study ASD subjects showed reduced intra-hemispheric as well as inter-hemispheric coherence in particular in the delta and theta band. Moreover they displayed lower inter-hemispheric coherences in the delta and theta bands in frontal regions, lower coherences in the delta, theta, and alpha bands in temporal regions and lower coherences in delta, theta, and beta bands in the central/parietal/occipital regions. The large amount of significant differences in coherence values in several brain regions, suggests altered connectivity in ASD (Belmonte et al., 2004).

Murias et al. (2007) have studied the eye-closed condition by using a high-density EEG system (124 electrodes) in adults with ASD. Indeed, a high-density approach, by employing more number of electrodes, allows increasing spatial resolution of the EEG potentials and improving signal source localization. A spectral analysis as well as a coherence analysis was performed. In this experiment, ASD group showed an elevated relative theta and reduced relative alpha power primarily in the frontal and prefrontal regions. In addition, a reduced relative alpha and increased relative beta power was observed in the occipital/parietal regions, with bilateral central regions approaching significance. Significant differences in coherence analysis between the two groups were also observed in theta and alpha bands. The results of this study are in agreement with the theory of local overconnectivity and global under-connectivity in ASD (Courchesne and Pierce, 2005). In fact EEG oscillations in the theta range reflect locally dominant neocortical processes, whereas alpha oscillations represent more globally dominant phenomena that are more dependent on cortico-cortical and callosal fibers (Nunez, 2006).

In a more recent study (Pop-Jordanova et al., 2010) both the open-eyes and the closed-eyes conditions were investigated. In this study EEG data obtained on ASD children were compared to data belonging to neurotypical subjects contained in a database. The authors found an increase delta/theta power in ASD in both conditions. Moreover they noticed that in the open-eyes condition there was an increase in beta power with respect to the closed-eyes condition in both groups. These results were only partially in agreement with the previous studies. The authors also have introduced here a new index, namely the spectrum weighted frequency (brain rate), as an indicator of general mental arousal in these subjects. They found a reduction of brain rate in all regions in autistic children compared to the controls, indicating a lower general mental arousal in ASD.

Also in the most recent study by Mathewson et al. (2012) where QEEG technique was applied in the study of high-functioning adults with ASD, both the open-eyes and the closed-eyes condition were analyzed. The novelty of this study is that the features extracted by QEEG analysis related to power and frequency, were correlated to behavioral performances measured with the Autism-Spectrum Quotient (AQ; Baron-Cohen et al., 2001). In particular in this study the scores obtained with this instrument were clustered in two groups: preferential attention to detail and deficits in social interaction. EEG data were continuously recorded by means of a 128-channel system during a resting baseline condition in which eyes-open and eyes-closed conditions alternated. The absolute PSD was calculated for each band separately at each single electrode, however, in order to reduce the computational complexity, power was averaged within four quadrants: left frontal, right frontal, left posterior, and right posterior. The main focus of the study was alpha band because it is thought to indirectly reflect the level of cortical excitability in the regions where it is found. It means that higher resting alpha power might denote cortical deactivation or inactivity (Rihs et al., 2007; Sauseng et al., 2009). Power analysis showed that in the eye-open condition the alpha power was higher in ASD group than in the controls in all regions, while in eye-closed condition no difference was found.

Regarding the other bands, beta, gamma, and theta powers were increased in ASD group. An analysis of the amount of alpha suppression in occipital regions was also performed. The results indicated that at O1 a significantly greater alpha suppression was present in control subjects than in ASD group. This difference in suppression is related to the fact that alpha suppression is associated with optimal neurological functioning that is impaired in ASD. In contradiction with the previous studies, no differences in coherence between the two groups were found.

While correlating the SP with the AQ score it was found that in ASD participants, alpha power was inversely correlated with preferential attention to detail in posterior and frontal regions both in eye-open and eye-closed condition, while no correlation was found in controls. From the coherence analysis, a positive correlation between alpha coherence in eye-closed condition and attention to details was found in the right centro-parietal region in controls, while in ASD group, attention to details was inversely correlated with coherence in eye-opened condition in the right centro-parietal region and in eye-closed condition in the parieto-occipital regions. Moreover in both groups alpha coherence in eye open was inversely correlated to social functioning in the right fronto-central region. For the other bands, increased theta coherence in the left centro-parietal region in eye-closed condition appeared to be related to poorer social functioning in ASD, while increased gamma coherence in the same region and condition appeared to be beneficial to social functioning in controls. The negative correlation between attention to detail and alpha power and coherence in parietal regions in ASD is consistent with the theory of altered parietal functioning in ASD. An explanation of this result could be that in ASD the mapping of attention focus may be exaggerated at the expense of attention prioritizing. Moreover the negative correlation between alpha coherence and attention to details is consistent with the fact that the brain is more receptive to incoming sensory information when neural activity is desynchronized than when it is engaged in performing a specific task.

In a few articles non-linear analysis techniques have been used to study particular properties of EEG signals. In the study of Sheikhani et al. (2007) the authors computed three types of transforms at each electrode for discriminating between ASD and controls: the STFT, the STFT-BW calculated considering the bandwidth of all the spectra as window, and the bispectrum. Data were acquired using a standard 10/20 system during eye-closed condition. The authors did not find any difference in STFT or bispectrum but they found significant differences in STFT-BW over Fp1, F3, F7, T3, T5, and O1. Since the sample of ASD and controls is very small in this study, it is difficult to draw any conclusions from the results. The bispectral analysis needs to be tested in a larger sample of subjects to see if it gives significant results and relevant information on brain activation not evidenced by the most common power spectral analysis.

In another study by Ahmadlou et al. (2010), the complexity of EEG signals in ASD and controls was investigated by using fractal dimension analysis. Data were acquired during eye-closed condition and a wavelet analysis approach aimed at decomposing the signal into the five standard EEG bands was performed. For each band and electrode the fractal dimension was computed using two methods: Higuchi’s Fractal Dimension (HFD) (Higuchi, 1988) and Katz’s Fractal Dimension (KFD) (Katz, 1988). Significant differences between ASD and controls in HFD were detected, especially in gamma, beta, and alpha and in KFD in gamma, beta, and delta. Differences were most significant for KFD, meaning that this method is a more effective tool for discriminating between ASD and controls. This study shows how fractal dimension, providing additional information about EEG signals, could be an important instrument for the identification of brain abnormalities in ASD.

Thatcher et al. (2009) applied phase analysis for the investigation of phase-reset mechanism in high-functioning children with ASD. Phase reset (PR) is defined as the succession of phase shifts (e.g., 30–80 ms) and phase locking (e.g., 100–800 ms) of clusters and sub-clusters of neurons. This process is regulated by GABA mediated thalamo-cortical circuits that is believed to be compromised in ASD (Orekhova et al., 2008). EEG data were acquired on ASD subjects and age-matched controls during eyes-open resting condition. Complex demodulation was used to compute phase differences between the signals from each pairs of electrodes. Each PR was composed by a phase shift of a finite duration (SD) and a phase locking of an extended duration (LD). SD is the interval of time from the onset of phase shift to its termination, defined by a peak in the first derivative and a peak in the second derivative or inflection on the declining side of the time series of first derivatives. LD was defined as the interval of time between the end of a significant phase shift and the beginning of a subsequent significant phase shift. Both in ASD and in controls SD, LD, and PR were computed for each pairs of electrodes in each EEG band. The comparison between the two groups showed that SD was significantly shorter in ASD than in the controls in particular in alpha-1 frequency band (8–10 Hz). On the contrary, LD was consistently longer in ASD especially in alpha-2 frequency band (10–12 Hz). The results of this study demonstrate an altered mechanism of neural synchronization in ASD, and suggest that increased phase lock periods, that represent the time in which clusters of neurons are synchronized, could reflect less cognitive flexibility and less availability of neural resources. However, higher statistically significant differences were found at short rather than long inter-electrode distances, suggesting that the defect in the mechanism of phase locking is particularly present in local neural.

In a recent study by Bosl et al. (2011) a non-linear analysis based on the calculation of MSE was performed. The analysis was applied to EEG signals acquired on a group of infants at “High Risk” because siblings of children with ASD. Data were obtained during resting state eyes open using a 64 channels EEG system. For a more accurate comparison, the sample set was divided in sub-groups according to age: 6, 9, 12, 18, and 24 months. The authors found a decrease of MSE in HRA over all EEG channels across all scales and ages. When considering the trajectories of mean MSE with age, it was shown that while the pattern of complexity was almost the same in the two groups in the age range 6–9 months, there was a strong decrease in EEG complexity in the HRA groups in the age range 9–12 months. Several classification approaches were then applied to distinguish between the groups of controls and HRA subjects according to the EEG complexity features. The machine learning approach represents an accurate classification method, especially in the first year of life of the children. As underlined by Griffin and Westbury (2011), in their commentary to the article of Bosl et al. (2011), these results should be viewed with caution because they identify an alteration of MSE in the whole population of HRA subjects without differentiating between the ones who will really develop autism and the ones who will have a typical development or other neuropsychiatric disorders. However, this study could be a promising starting point to carry out further investigation in order to highlight the differences in EEG complexity at the individual level, possibly helping to predict the risk of developing autistic traits.

Another interesting attempt in differentiating ASD and controls group based on a data-driven approach has been recently performed by Duffy and Als (2012). In this study, EEG data were recording on a large sample of ASD and TD subjects during awake state and coherence values were measured for each EEG frequency band.

In a first step, the dimensionality of the coherence data was reduced by applying a Principal Component Analysis (PCA) based on a Singular Value Decomposition (SVD) approach (SVD-based PCA). In the following step a classification algorithm, in particular the Discriminant Function Analysis (DFA), was applied to the variables selected by PCA. This technique allows producing a new canonical variable, the discriminant function, which is based on a weighted combination of the input variables and allows maximally separating the ASD and TD groups.

Given the wide age range of subjects, the classification analysis was applied also to subset of subjects with a narrower age span. The coherence factors given by DFA analysis allowed to accurately classifying ASD and TD across all three age spans. Moreover, it was observed that 70% of the factors were associated with reduced coherence for ASD subjects, in particular in the left temporal regions and frontal (short–distance connections). The decreased connectivity within these regions could be associated to language and communication problems. The other coherence factors showed an increased coherence in ASD subjects in particular in the long-distance connection. This finding could be explained as a compensatory mechanism of the autistic brain which establish atypical, spatially disparate, cortical networks to replace deficit function normally associated to more localized network. These results were quite stable across all broad spectral ranges.

The results of this study are very encouraging because it seems that the coherence factors could be used as a possible useful neurophysiological ASD-phenotype.

### QEEG during specific tasks

The application of QEEG processing technique during cognitive tasks can give the possibility to view the dynamic changes which take place in the brain during these conditions, determining in this way which areas of the brain are engaged. Although in the past some researchers have considered that EEG signals acquired during task conditions are destabilized or otherwise corrupted (Thatcher, 1998), recent researches have challenged this conclusion. For example, McEvoy et al. (2000) have demonstrated greater stability of QEEG signals recorded during cognitive tasks, with respect to the resting condition and therefore paving the way for developing task-specific understandings of brain operation.

Oberman et al. (2005) have analyzed the mu (8–13 Hz) power, an index of neuron synchronization or desynchronization, over the sensorimotor cortex during imitation tasks. At rest, sensorimotor neurons spontaneously fire in synchrony, leading to large amplitude EEG oscillations and to elevated power in mu frequency band. Conversely, during action, these neurons fire asynchronously, and therefore the power in mu band decreases. In this investigation, EEG from high-functioning ASD and controls were recorded during observation of biological and non-biological motion. While controls showed a decreased mu power both in self-initiated hand movement and in observed biological motion conditions, the ASD group obtained the same effect only during the first task, suggesting mirror neuron dysfunction in autism.

In fact, sensorimotor neurons could be considered as belonging to the well-known mirror neurons system (Rizzolatti et al., 2001). Several studies have related the imitation deficit in subjects with autism to an impairment of this neural circuitry (Williams et al., 2001; Nishitani et al., 2004; Iacoboni and Dapretto, 2006). Nevertheless, the findings of some recent studies argue against a mirror system dysfunction in ASD (Dinstein et al., 2010; Fan et al., 2010).

Sheikhani et al. (2012) used the spectrogram method to analyze data acquired on a group of children with ASD and age-matched controls during sustained visual attention. The spectrogram criteria – defined as the average of all the frequency component values of spectrogram >70% of the maximum value for each frequency band – as well as the coherence between pairs of different electrodes were computed. The ASD group exhibited lower values of spectrogram criteria in alpha, beta, and gamma bands, whereas no significant difference was observed in the delta band. According to these results, EEG signal show the most significant differences in the temporal and in the frontal regions of the left brain hemisphere. These results agree with several studies showing an impairment of left hemisphere, in particular in temporal and frontal regions, in ASD (Rojas et al., 2002, 2005; Chandana et al., 2005). The authors also showed an increase in the degree of coherence in the ASD group, and suggested increased functional connectivity of temporal lobes with other regions in the gamma band frequency.

In another investigation of the same authors (Sheikhani et al., 2009), children with ASD and controls underwent EEG acquisition in nine different conditions: [(1) eye-closed condition, (2) eye-opened condition, (3–5) looking at three samples of Kanizsa shapes, (6) looking at mother’s picture upright and (7) inverted, (8) looking at stranger’s picture upright, and (9) inverted in frequency bands]. Spectrogram and PSDs were calculated for each band at each condition. In the relaxed eye-opened condition, children with ASD obtained significant differences in gamma band with lower values of spectrogram criteria and higher values of SP. Spectrogram criteria were also significantly different in the alpha band when ASD and control children looked at the inverted mother’s picture, and in alpha, beta, and gamma bands, when they looked at an inverted stranger’s picture. Given that gamma band seems to play a role in the synchronization of cortical nets region, especially in recognition and perception, the authors suggested an abnormal functioning on these issues in ASD. Furthermore, since the alpha band is associated with the coordination of wider areas of the brain, and beta band plays a role in integrating neighboring areas, the abnormal spectrogram criteria found in this study might suggest a defect of coordination and integration in ASD.

In a recent study (Chan et al., 2011a), QEEG techniques were employed in order to examine the association between memory performance and fronto-posterior theta coherence in individuals with ASD. Several studies have found associations of theta-band amplitude with the performance of working memory tasks (Klimesch et al., 1994) and long-term memory encoding and retrieval (Larson et al., 1986). Moreover, basic research in animals and neuroimaging studies in humans have shown that during working memory tasks multiple brain areas are activated, in particular the prefrontal and postrolandic association cortices as well as the cingulate cortex and medial temporal areas (Fuster, 1995; Krause et al., 2000; Postle and D’Esposito, 2000). Given these findings, it has been suggested that connectivity abnormalities in these brain areas could be the neural bases of memory deficits in autism (Rippon et al., 2007). In the study by Chan et al. (2011a), EEG data were recorded during an object recognition task. ASD individuals showed elevated fronto-posterior long-range theta coherences, both intra-hemispheric (in the left hemisphere) and inter hemispheric (from left anterior to right posterior regions). Moreover, an opposite asymmetry pattern was observed: coherences in controls were higher in the right than in the left hemisphere, while in ASD children the pattern was opposite. A significant negative correlation between memory performance and inter-hemispheric long-range coherence was present in ASD subjects, whilst no significant correlations were found in controls. The abnormal pattern in ASD children could be explained with a hyper-functional connectivity in theta band with respect to controls that decrease the efficiency of memory processing.

In another study by the same research group (Chan et al., 2011b) the association between the performance of children with ASD in attention and inhibitory control and brain activity was investigated. The analysis was focused on relative PSD within theta band, which is related to attentional and inhibitory processing during a Go/No-Go task.

The authors found a decrease of theta activity in ASD children with respect to controls in the anterior regions for the “Go” and in anterior and centrotemporal regions for the “No-Go” condition. The application of LORETA software allowed a more accurate source localization ad it showed that in the “Go” condition theta decreased occurred in particular in the anterior cingulate cortex (ACC), while in the “No-Go” condition in the ACC and also in the precuneus. Significant correlations were found between theta power and scores obtained at the several tests performed showing an association between depressed brain activity, in particular in the ACC, and poorer performance in attention and inhibition.

Some authors have outlined the importance of studying the high EEG frequencies in order to characterize brain activity in ASD. The paper from Orekhova et al. (2007) aims at analyzing the differences between controls and ASD in high frequency EEG bands. EEG activity was recorded in young children with autism and age-matched controls during sustained visual attention. The mean PSD was calculated in three high frequency bands: beta (13.2–24 Hz), gamma 1 (24.4–44.0 Hz), and gamma 2 (56.0–70 Hz). An enhancement of spontaneous high frequency EEG oscillations in ASD was found, especially in gamma 1 band. The most involved brain areas were the midline, central, and parietal regions. Moreover, a significant positive correlation between the power spectrum value of gamma 1 and the degree of developmental delay in ASD group was detected. The excess of high frequencies in ASD agrees with the theory of an increase in ratio of excitation/inhibition in autism that leads to the formation of “noisy” and unstable cortical networks (Rubenstein and Merzenich, 2003).

In the most recent study (Lushchekina et al., 2012), the authors have tried to identify the neurophysiological components of cognitive abnormalities in ASD. EEG recordings, made in the standard 10/20 scheme were performed at baseline (rest with closed eyes), and during a cognitive task, consisting of counting, adding, and subtracting. SP and mean coherence were studied in the alpha, beta, and gamma ranges. Both typical and ASD subjects showed a marked frontal-occipital alpha gradient in baseline conditions. ASD individuals were characterized by right-sided predominance of PSD in the alpha range, both at rest and during cognitive tasks. In addition, in ASD the PSD of the gamma rhythm in baseline conditions was higher than that in the controls. During the cognitive task in ASD group the SP and mean coherence of fast rhythms did not change.

Another study (Catarino et al., 2011) analyzed EEG data acquired from adult ASD individuals and controls during a visual task (pictures of neutral faces versus pictures of chairs); a complexity analysis was performed using the MSE measure already described (Bosl et al., 2011). The task consisted in the detection of pictures of neutral faces and of chairs. Both a MSE investigation and a more traditional power spectral analysis were performed for each group and condition. While no differences were found in PSD, the authors demonstrated reduced entropy in ASD with respect to controls, especially at higher time scales, confirming that the decrease of MSE can be associated to impairments in brain function and connectivity.

### QEEG for the identification of autistic subgroups

Clinical observation as well as research data, suggest that ASD are a set of neurodevelopmental disorders with a considerable heterogeneity in the phenotypic presentation (Witwer and Lecavalier, 2008; Georgiades et al., 2013). Among the several methods used to stratify ASD subjects into more homogeneous subgroups, the QEEG may provide more objective and quantitative features characterizing different groups of affected individuals. However, despite the fact that QEEG approach seems very promising, only few studies have so far been directed to this end.

The first investigation on this topic (Dawson et al., 1995) showed how QEEG is not only useful in differentiating subjects with high-functioning ASD from controls, but also in distinguishing amongst subgroups which differ in degree and nature of social impairments. Twenty-eight children with autism were classified according to Wing and Gould (1979) classification system: “Aloof,” “Passive,” and “Active-but-odd.” In particular, the authors studied the “passive” and the “active-but-odd” groups. EEG was acquired during sustained visual attention, and the PSD were calculated for each band. The passive group showed reduced EEG power in delta and theta bands in all brain regions and reduced alpha power in the frontal regions. Since the alpha activity is related to social engagement, a reduced alpha activity could reflect, in this “passive” group, a lack of active engagement in social information processing.

A subsequent study by Sutton et al. (2005), QEEG analysis was performed in order to correlate resting cortical brain activity with social-emotional abilities behaviors in high functioning children with autism (HFA).

Data were acquired on high-functioning ASD children and controls during eyes-opened and closed conditions. The analysis focused on the alpha band, due to its stronger relation with behavioral measures with respect to other frequency bands. Moreover an asymmetry index was calculated for homologous electrode pairs and was used for subgrouping autistic children. Three subgroups were obtained using computer-generated cut points: (1) the most extreme right mid-frontal asymmetry scores (RFA group), (2) the most extreme left medial frontal asymmetry scores (LFA group), and (3) the intermediate frontal asymmetry (IFA group A reduction of alpha power density in anterior, central, and posterior cortical regions of control individuals compared with HFA subjects was detected. Moreover, the HFA group showed a different asymmetry pattern compared to the control group. Finally, some brain-behavior relationship were found: in particular, the LFA group reported greater symptoms of anxiety and social stress, while the RFA group was characterized by a greater social impairment.

## Discussion and Conclusion

This review focuses on key findings of quantitative EEG application in subjects with ASD. Despite conflicting results, literature analysis suggests that QEEG may help in detecting features of altered brain function, in linking behavior with brain activity and in defining more phenotypically homogeneous subgroups within the affected individuals.

Taken together, reviewed studies show that children with ASD present several differences in power spectra, coherence, and symmetry measures with respect to controls. This is true both when the signals are acquired in resting conditions – with either open or closed eyes – and when specific tasks are performed. However, QEEG features strongly depend on the diverse experimental settings (for example EEG recorded during observation of actions or during execution of actions) that may lead to different results. In addition, most parameters such as power spectra, coherence, and asymmetry, change with age and may vary according to behavioral, cognitive, and comorbid features of ASD subjects. The wide heterogeneity of the samples examined in the literature, particularly with regard to the cognitive level and age of subjects, and the different criteria used to diagnose ASD, makes it difficult to compare these studies and achieve unique general conclusions. In addition, drugs could influence the EEG activity (Muroka et al., 1992; Banoczi, 2005) with a potential impact on brain developmental process, especially in the frontal regions, which are the slowest in maturating. While some studies are more restrictive in defining the exclusion criteria of the sample, avoiding the enrollment of subjects taking medication, others do not define strict exclusion/inclusion criteria, or at least there are not clearly mentioned in the participant description. Thus, the variability in medication use across studies may be responsible for mixed findings in the literature reviewed. A possible role of immune dysregulation, toxicant exposures, and metabolic factors on the development of ASD abnormalities has been suggested (for a recent review, see Rossignol and Frye, 2012). However, the evaluation of these issues are often not specified or considered in the studies examined, potentially accounting for variation across studies and within subjects.

In open-eyes condition, the differences between ASD and TD are more pronounced. Studies performed in an eye-open rest condition present some replicated finding, i.e., the constant increase on delta power in ASD with respect to controls and the decrease in high frequency, especially alpha in childhood and adolescents (Cantor et al., 1986; Chan et al., 2007; Stroganova et al., 2007; Pop-Jordanova et al., 2010), but also some contradictory results. The power of alpha band in ASD, with respect to typical controls, was found to be reduced (Cantor et al., 1986; Chan et al., 2007), unchanged (Stroganova et al., 2007), or even increased (Mathewson et al., 2012) in different studies. A greater level of alpha amplitude reflects the inhibition of non-essential activity and consequently a better performance on the task (Klimesch et al., 2007) that could be explained by the *neural efficiency hypothesis* (Doppelmayr et al., 1998).

Also, the degree of asymmetry was found either broad-band decreased in ASD (Cantor et al., 1986) or leftward increased (Stroganova et al., 2007). This latter study also showed a symmetric mu rhythm, which was paradoxically asymmetric in healthy controls. The analysis of coherence between and within hemispheres in ASD subjects revealed an increased (Cantor et al., 1986) or reduced finding (Mathewson et al., 2012).

Several demographic and clinical differences characterize the samples involved in the above-mentioned studies, which may in part explain the conflicting results. In particular, the age range varies among the studies considered: young children (Cantor et al., 1986; Stroganova et al., 2007) children and adolescents (Chan et al., 2007), and adults (Mathewson et al., 2012) were respectively enrolled. Moreover, the cognitive level of ASD children displays a wide range of abilities: low functioning (Cantor et al., 1986), both high and low functioning (Stroganova et al., 2007), and only high-functioning (Chan et al., 2007; Mathewson et al., 2012) ASD subjects were evaluated.

Since QEEG indices are related to brain maturation and development (Clarke et al., 2001), the age represents a critical factor in the interpretation of results.

In typical development children low frequencies tend to decrease with age from childhood to adulthood while high frequencies increase (Gasser et al., 1988a). As regards coherence it broad-band increases with age (Gasser et al., 1988b). Autistic children seems to have a late maturation as they show more slow-wave and less alpha activity, as well as greater coherence than the age-matched typical controls. As coherence increases with age in TD subjects, in adulthood coherence values became comparable between ASD and TD groups (Murias et al., 2007; Mathewson et al., 2012).

In addition, EEG activity is influenced by level of cognitive abilities: in fact, Thatcher et al. (2005) showed that absolute EEG power is positively correlated with full scale, verbal, and performance IQ, while coherence is negatively correlated with these scores.

The comorbidity between ASD and other psychiatric or neurological disorders is a common feature of ASD clinical manifestation (Simonoff et al., 2008). The presence of an additional non-ASD disorder represents a potential confounding factor in EEG research that frequently hasn’t been taken into account in the interpretation of results. Thus, future exploration into the EEG presentations of subjects with comorbid psychopathology versus ASD singly may be of seminal importance for a better knowledge of the ASD biological underpinnings.

In closed-eyes condition, results are even more contradictory both in terms of power and coherence (Murias et al., 2007; Coben et al., 2008; Pop-Jordanova et al., 2010; Mathewson et al., 2012). Except for the study of Pop-Jordanova et al. (2010), which do not define the cognitive level, all these studies enrolled high-functioning individuals. In addition, two studies (Murias et al., 2007; Mathewson et al., 2012) have been performed on adults, whereas one investigation (Coben et al., 2008) on scholar children and another (Pop-Jordanova et al., 2010) on pre-scholar children. This suggests again that the different age range of the subjects participating to the different studies may at least in part influence the results. Moreover, the different findings of Murias et al. (2007) and Mathewson et al. (2012) on adult subjects (with respect to both alpha power and coherence) may in part be due to the different methodological approach. By considering all the 128 electrodes in computing power spectrum and coherence, both a decrease in power and coherence in alpha band (Murias et al., 2007), and no differences (Mathewson et al., 2012) were detected. Therefore, the analysis of neural networks with higher spatial resolution seems to allow a thinner characterization of brain activation and connectivity. Source localization using software like LORETA used in the study by Chan et al. (2011b) can also be useful to increase the spatial resolution of EEG.

In closed-eyes condition the increase in slow-way activity is more related to theta than delta. Moreover there is an increase in beta frequencies from childhood to adulthood, which is not observed in open-eyes condition (Murias et al., 2007; Coben et al., 2008). Increase of beta activity is associated with a strengthening of sensory feedback in static motor control when movement has to be resisted or voluntarily suppressed (Lalo et al., 2007; Zhang et al., 2008). In children with ASD the increased beta activity in closed-eyes condition may reflect the difficulty in motor and sensorial regulation that they present in this situation.

With regards to coherence, it seems that increases with age such as controls: in fact, it is decreased in childhood and adolescence and become equal or increased in adulthood.

Although the exact meaning of changes in SP and coherence in ASD children is not easy to understand, in resting state condition, both dysfunction of general state of arousal or of more specific systems of cognitive processing may explain these findings. However, by correlating brain activity findings with behavioral measures, Mathewson et al. (2012) showed that cognitive function and modulation might influence QEEG also at rest.

Acquiring data while children perform specific tasks allows having a better characterization of the link between behavior and brain activation, although the possibility to drive definitive conclusions is limited due to the small number of studies and of sample size. Differences between ASD subjects and controls during tasks mainly involve high frequencies, alpha, beta, and gamma, which have been found increased (Orekhova et al., 2007; Sheikhani et al., 2009; Lushchekina et al., 2012) in ASD population, regardless of the type of task. Moreover some authors also found an increase in coherence during tasks, in ASD with respect to control, supporting the hypothesis of an enhanced functional connection between cortical networks (overconnectivity) at the basis of the aberrant behaviors observed in autism.

In literature, moreover, QEEG was used for subtyping ASD subjects (Dawson et al., 1995; Sutton et al., 2005), suggesting that some QEEG parameters may correlate with different behavioral phenotype The identification of subgroups of subjects with different QEEG profiles could contribute to increase the homogeneity of ASD samples, with the aim to detect specific developmental time course, treatment responses, and possibly pathophysiological underpinnings.

In addition, due to the fact that brain activation and QEEG measures are strictly dependent on age, it is very important evaluating developmental processes in autism. QEEG, in fact, may show different developmental patterns in infants with high and low risk for ASD, and could be therefore used as a promising endophenotype for early diagnosis in at-risk children (Tierney et al., 2012). Non-linear techniques, such as entropy (Bosl et al., 2011) have also been used at this end. This technique, like also fractal dimension or phase coupling (Sheikhani et al., 2007; Thatcher et al., 2009; Ahmadlou et al., 2010; Bosl et al., 2011), is appealing not only in order to characterize autistic brain, but also to obtain potential biomarkers of the disorder, not otherwise detectable with common linear methods.

Overall, it is important to underline that QEEG activity components may also have some individual characteristics that differentiate each subject. The assessment of these features has a crucial importance for establishing a QEEG “baseline,” which may be different for each person.

New advanced analysis methods such as entropy or cluster analysis could be useful to identify autistic subgroups with specific neurophysiological characteristics, providing in this way different brain endophenotypes, which may benefit from different intervention strategies. Thus, this sort of metrics on the brain’s function could be used, in the future, to develop personalized treatments (for example by using connectivity-guided neurofeedback), and evaluate the effects of therapies through quantitative measures of brain activity. Also in this case, source localization is extremely important: in fact, variation of brain activity in a specific brain area can be a quick and objective indicator to monitor the effect of the treatment.

## Conflict of Interest Statement

The authors declare that the research was conducted in the absence of any commercial or financial relationships that could be construed as a potential conflict of interest.

## References

[B1] Ahmadlou M. Adeli H. Adeli A. (2010). Fractality and a wavelet-chaos-neural network methodology for EEG-based diagnosis of autistic spectrum disorder. J. Clin. Neurophysiol. 27, 328–333 10.1097/WNP.0b013e3181f40dc8 20844443

[B2] American Psychiatric Association (2013). Diagnostic and Statistical Manual of Mental Disorders, 5th Edn. Washington, DC: American Psychiatric Association

[B3] Banoczi W. (2005). How some drugs affect the electroencephalogram (EEG). Am. J. Electroneurodiagnostic Technol. 45, 118–129 15989074

[B4] Barnea-Goraly N. Kwon H. Menon V. Eliez S. Lotspeich L. Reiss A. L. (2004). White matter structure in autism: preliminary evidence from diffusion tensor imaging. Biol. Psychiatry 55, 323–326 10.1016/j.biopsych.2003.10.022 14744477

[B5] Barnier R. Dawson G. Webb S. Murias M. (2007). EEG mu rhythm and imitation impairments in individuals with autism spectrum disorder. Brain Cogn. 64, 228–237 10.1016/j.bandc.2007.03.004 17451856 PMC2709976

[B6] Baron-Cohen S. Wheelwright S. Skinner R. Martin J. Clubley E. (2001). The autism-spectrum quotient (AQ): evidence from Asperger syndrome/high-functioning autism, males and females, scientists and mathematicians. J. Autism Dev. Disord. 31, 5–17 10.1023/A:1005653411471 11439754

[B7] Belmonte M. K. Allen G. Beckel-Mitchener A. Boulanger L. M. Carper R. A. Webb S. J. (2004). Autism and abnormal development of brain connectivity. J. Neurosci. 24, 9228–9231 10.1523/JNEUROSCI.3340-04.2004 15496656 PMC6730085

[B8] Belmonte M. K. Yurgelun-Todd D. A. (2003). Functional anatomy of impaired selective attention and compensatory processing in autism. Brain Res. Cogn. Brain Res. 17, 651–664 10.1016/S0926-6410(03)00189-7 14561452

[B9] Billeci L. Calderoni S. Tosetti M. Catani M. Muratori F. (2012). White matter connectivity in children with autism spectrum disorders: a tract-based spatial statistics study. BMC Neurol. 12:148 10.1186/1471-2377-12-148 23194030 PMC3607981

[B10] Boddaert N. Chabane N. Gervais H. Good C. D. Bourgeois M. Plumet M.-H. (2004). Superior temporal sulcus anatomical abnormalities in childhood autism: a voxel-based morphometry MRI study. Neuroimage 23, 364–369 10.1016/j.neuroimage.2004.06.016 15325384

[B11] Bosl W. Tierney A. Tager-Flusberg H. Nelson C. (2011). EEG complexity as a biomarker for autism spectrum disorder risk. BMC Med. 9:18 10.1186/1741-7015-9-18 21342500 PMC3050760

[B12] Bourgeron T. (2009). A synaptic trek to autism. Curr. Opin. Neurobiol. 19, 231–234 10.1016/j.conb.2009.06.003 19545994

[B13] Calderoni S. Retico A. Biagi L. Tancredi R. Muratori F. Tosetti M. (2012). Female children with autism spectrum disorder: an insight from mass-univariate and pattern classification analyses. Neuroimage 59, 1013–1022 10.1016/j.neuroimage.2011.08.070 21896334

[B14] Cantor D. S. Thatcher R. W. Hrybyk M. Kaye H. (1986). Computerized EEG analyses of autistic children. J. Autism Dev. Disord. 16, 169–187 10.1007/BF01531728 3722118

[B15] Catarino A. Churches O. Baron-Cohen S. Andrade A. Ring H. (2011). Atypical EEG complexity in autism spectrum conditions: a multiscale entropy analysis. Clin. Neurophysiol. 122, 2375–2383 10.1016/j.clinph.2011.05.004 21641861

[B16] Centers for Disease Control and Prevention (2012). Prevalence of autism spectrum disorders – autism and developmental disabilities monitoring network, 14 sites, United States, 2008. MMWR Surveill. Summ. 61, 1–19 22456193

[B17] Chan A. S. Han Y. M. Y. Sze S. L. Cheung M. Leung W. W. Chan R. C. K. (2011a). Disordered connectivity associated with memory deficits in children with autism spectrum disorders. Res. Autism Spectr. Disord. 5, 237–245 10.1016/j.rasd.2010.04.007

[B18] Chan A. S. Han Y. M. Y. Sze S. L. Cheung M. Leung W. W. Leung C. (2011b). Abnormalities in the anterior cingulate cortex associated with attentional and inhibitory control deficits: a neurophysiological study on children with autism spectrum disorders. Res. Autism Spectr. Disord. 5, 254–266 10.1016/j.rasd.2010.04.007

[B19] Chan A. S. Sze S. L. Cheung M.-C. (2007). Quantitative electroencephalographic profiles for children with autistic spectrum disorder. Neuropsychology 21, 74–81 10.1037/0894-4105.21.1.74 17201531

[B20] Chandana S. R. Behen M. E. Juhász C. Muzik O. Rothermel R. D. Mangner T. J. (2005). Significance of abnormalities in developmental trajectory and asymmetry of cortical serotonin synthesis in autism. Int. J. Dev. Neurosci. 23, 171–182 10.1016/j.ijdevneu.2004.08.002 15749243

[B21] Clarke A. R. Barry R. J. McCarthy R. Selikowitz M. (2001). Age and sex effects in the EEG: development of the normal child. Neurophysiol. Clin. 112, 806–814 10.1016/S1388-2457(01)00488-6 11336896

[B22] Coben R. Clarke A. R. Hudspeth W. Barry R. J. (2008). EEG power and coherence in autistic spectrum disorder. Clin. Neurophysiol. 119, 1002–1009 10.1016/j.clinph.2008.01.013 18331812

[B23] Coben R. Linden M. Myers T. E. (2010). Neurofeedback for autistic spectrum disorder: a review of the literature. Appl. Psychophysiol. Biofeedback 35, 83–105 10.1007/s10484-009-9117-y 19856096

[B24] Costa M. Goldberger A. L. Peng C. K. (2002). Multiscale entropy analysis of complex physiologic time series. Phys. Rev. Lett. 89, 068102 10.1103/PhysRevLett.89.068102 12190613

[B25] Courchesne E. Pierce K. (2005). Why the frontal cortex in autism might be talking only to itself: local over-connectivity but long-distance disconnection. Curr. Opin. Neurobiol. 15, 225–230 10.1016/j.conb.2005.03.001 15831407

[B26] Courchesne E. Pierce K. Schumann C. M. Redcay E. Buckwalter J. A. Kennedy D. P. (2007). Mapping early brain development in autism. Neuron 56, 399–413 10.1016/j.neuron.2007.10.016 17964254

[B27] Dawson G. Klinger L. G. Panagiotides H. Lewy A. Castelloe P. (1995). Subgroups of autistic children based on social behavior display distinct patterns of brain activity. J. Abnorm. Child Psychol. 23, 569–583 10.1007/BF01447662 8568080

[B28] Dinstein I. Thomas C. Humphreys K. Minshew N. Behrmann M. Heeger D. J. (2010). Normal movement selectivity in autism. Neuron 66, 461–469 10.1016/j.neuron.2010.03.034 20471358 PMC2872627

[B29] Doppelmayr M. Klimesch W. Pachinger T. Rippe B. (1998). The functional significance of absolute power with respect to event-related desynchronization. Brain Topogr. 11, 133–140 10.1023/A:1022206622348 9880171

[B30] Duffy F. H. Als H. (2012). A stable pattern of EEG spectral coherence distinguishes children with autism from neurotypical controls – a large case control study. BMC Med. 10:64 10.1186/1741-7015-10-64 22730909 PMC3391175

[B31] Dumermuth G. Molinari L. (1987). Spectral analysis of the EEG: some fundamentals revisited and some open problems. Neuropsychobiology 17, 85–99 10.1159/000118345 3306442

[B32] Fan Y. T. Decety J. Yang C. Y. Liu J. L. Cheng Y. (2010). Unbroken mirror neurons in autism spectrum disorders. J. Child Psychol. Psychiatry 51, 981–988 10.1111/j.1469-7610.2010.02269.x 20524939

[B33] Fries P. (2005). A mechanism for cognitive dynamics: neuronal communication through neuronal coherence. Trends Cogn. Sci. (Regul. Ed.) 9, 474–480 10.1016/j.tics.2005.08.011 16150631

[B34] Fuster J. M. (1995). Memory in the Cerebral Cortex – An Empirical Approach to Neural Networks in the Human and Nonhuman Primate. Cambridge, MA: MIT Press

[B35] Gasser T. Verleger R. Bacher P. Sroka L. (1988a). Development of the EEG of school-age children and adolescents. I. Analysis of band power. Electroencephalogr. Clin. Neurophysiol. 69, 91–99 10.1016/0013-4694(88)90204-0 2446839

[B36] Gasser T. Verleger R. Bacher P. Sroka L. (1988b). Development of the EEG of school-age children and adolescents. II. Topography. Electroencephalogr. Clin. Neurophysiol. 69, 100–109 10.1016/0013-4694(88)90205-2 2446829

[B37] Georgiades S. Szatmari P. Boyle M. Hanna S. Duku E. Zwaigenbaum L. (2013). Investigating phenotypic heterogeneity in children with autism spectrum disorder: a factor mixture modeling approach. J. Child Psychol. Psychiatry 54, 206–215 10.1111/j.1469-7610.2012.02588.x 22862778

[B38] Griffin R. Westbury C. (2011). Infant EEG activity as a biomarker for autism: a promising approach or a false promise? BMC Med. 9:61 10.1186/1741-7015-9-61 21599952 PMC3117727

[B39] Higuchi T. (1988). Approach to an irregular time series on the basis of the fractal theory. Physica D 31, 277–283 10.1016/0167-2789(88)90081-4

[B40] Hubl D. Bölte S. Feineis-Matthews S. Lanfermann H. Federspiel A. Strik W. (2003). Functional imbalance of visual pathways indicates alternative face processing strategies in autism. Neurology 61, 1232–1237 10.1212/01.WNL.0000091862.22033.1A 14610126

[B41] Hugdahl K. (1996). Brain laterality – beyond the basics. Eur. Psychol. 3, 206–220 10.1027/1016-9040.1.3.206

[B42] Hughes J. R. John E. R. (1999). Conventional and quantitative electroencephalography in psychiatry. J. Neuropsychiatry Clin. Neurosci. 11, 190–208 10333991 10.1176/jnp.11.2.190

[B43] Iacoboni M. Dapretto M. (2006). The mirror neuron system and the consequences of its dysfunction. Nat. Rev. Neurosci. 7, 942–951 10.1038/nrn2024 17115076

[B44] John E. R. Karmel B. Z. Corning W. C. Easton P. Brown D. Ahn H. (1977). Neurometrics. Science 196, 1393–1410 10.1126/science.867036 867036

[B45] Katz M. J. (1988). Fractals and the analysis of waveforms. Comput. Biol. Med. 18, 145–156 10.1016/0010-4825(88)90041-8 3396335

[B46] Klimesch W. Sauseng P. Hanslmayr S. (2007). EEG alpha oscillations: the inhibition-timing hypothesis. Brain Res. Rev. 53, 63–88 10.1016/j.brainresrev.2006.06.003 16887192

[B47] Klimesch W. Schimke H. Schwaiger J. (1994). Episodic and semantic memory: an analysis in the EEG theta and alpha band. Electroencephalogr. Clin. Neurophysiol. 91, 428–441 10.1016/0013-4694(94)90164-3 7529682

[B48] Koenig T. Prichep L. Lehmann D. Sosa P. V. Braeker E. Kleinlogel H. (2002). Millisecond by millisecond, year by year: normative EEG microstates and developmental stages. Neuroimage 16, 41–48 10.1006/nimg.2002.1070 11969316

[B49] Kouijzer M. E. J. de Moor J. M. H. Gerrits B. J. L. Congedo M. van Schie H. T. (2010). Neurofeedback improves executive functioning in children with autism spectrum disorders. Res. Autism Spectr. Disord. 3, 145–162 10.1016/j.rasd.2008.05.001

[B50] Kouijzer M. E. J. van Schie H. T. de Moor J. M. H. Gerrits B. J. L. Buitelaar J. K. (2009). Neurofeedback treatment in autism. Preliminary findings in behavioral, cognitive, and neurophysiological functioning. Res. Autism Spectr. Disord. 4, 386–399 10.1016/j.rasd.2009.10.007

[B51] Krause C. M. Sillanmäki L. Koivisto M. Saarela C. Häggqvist A. Laine M. (2000). The effects of memory load on event-related EEG desynchronization and synchronization. Clin. Neurophysiol. 111, 2071–2078 10.1016/S1388-2457(00)00429-6 11068244

[B52] Lachaux J. P. Rodriguez E. Martinerie J. Varela F. J. (1999). Measuring phase synchrony in brain signals. Hum. Brain Mapp. 8, 194–208 10.1002/(SICI)1097-0193(1999)8:4<194::AID-HBM4>3.0.CO;2-C 10619414 PMC6873296

[B53] Lalo E. Gilbertson T. Doyle L. Di Lazzaro V. Cioni B. Brown P. (2007). Phasic increases in cortical beta activity are associated with alterations in sensory processing in the human. Exp. Brain Res. 177, 137–145 10.1007/s00221-006-0828-5 16972074

[B54] Larson J. Wong D. Lynch G. (1986). Patterned stimulation at the theta frequency is optimal for the induction of hippocampal long-term potentiation. Brain Res. 368, 347–350 10.1016/0006-8993(86)90579-2 3697730

[B55] Lehmann D. (1990). “Brain electric microstates and cognition: the atoms of thought,” in Machinery of the Mind, ed. John E. R. (Boston: Birkhauser), 209–224

[B56] Lehmann D. Ozaki H. Pal I. (1987). EEG alpha map series: brain micro-states by space-oriented adaptive segmentation. Electroencephalogr. Clin. Neurophysiol. 67, 271–288 10.1016/0013-4694(87)90025-3 2441961

[B57] Lushchekina E. A. Podreznaya E. D. Lushchekina V. S. Strelets V. B. (2012). A Comparison EEG study in normal and autistic children. Neurosci. Behav. Physiol. 42, 236–243 10.1007/s11055-012-9558-2

[B58] Mandelbrot B. B. (1977). The Fractal Geometry of Nature. New York: Freeman and Company

[B59] Mathewson K. J. Jetha M. K. Drmic I. E. Bryson S. E. Goldberg J. O. Schmidt L. A. (2012). Regional EEG alpha power, coherence, and behavioral symptomatology in autism spectrum disorder. Clin. Neurophysiol. 123, 1798–1809 10.1016/j.clinph.2012.02.061 22405935

[B60] McAlonan G. M. Cheung V. Cheung C. Suckling J. Lam G. Y. Tai K. S. (2005). Mapping the brain in autism. A voxel-based MRI study of volumetric differences and intercorrelations in autism. Brain 128, 268–276 10.1093/brain/awh332 15548557

[B61] McEvoy L. K. Smith M. E. Gevins A. (2000). Test-retest reliability of cognitive EEG. Clin. Neurophysiol. 111, 457–463 10.1016/S1388-2457(99)00258-8 10699407

[B62] Muratori F. Calderoni S. Apicella F. Filippi T. Santocchi E. Calugi S. (2012). Tracing back to the onset of abnormal head circumference growth in children with autism spectrum disorder. Res. Autism Spectr. Disord. 6, 442–449 10.1016/j.rasd.2011.07.004

[B63] Murias M. Webb S. J. Greenson J. Dawson G. (2007). Resting state cortical connectivity reflected in EEG coherence in individuals with autism. Biol. Psychiatry 62, 270–273 10.1016/j.biopsych.2006.11.012 17336944 PMC2001237

[B64] Muroka M. Tada K. Nogami Y. Ishikawa K. Nagoya T. (1992). Residual effects of repeated administration of triazolam and nitrazepam in healthy volunteers. Neuropsychobiology 25, 134–139 10.1159/000118823 1407479

[B65] Narzisi A. Muratori F. Calderoni S. Fabbro F. Urgesi C. (2012). Neuropsychological profile in high functioning autism spectrum disorders. J. Autism Dev. Disord. 43, 1895–1909 10.1007/s10803-012-1736-0 23224514

[B66] Nishitani N. Avikainen S. Hari R. (2004). Abnormal imitation-related cortical activation sequences in Asperger’s syndrome. Ann. Neurol. 55, 558–562 10.1002/ana.20031 15048895

[B67] Nunez P. L. (2006). Neocortical Dynamics and Human EEG Rhythms. New York: Oxford University Press

[B68] Nuwer M. (1997). Assessment of digital EEG, quantitative EEG, and EEG brain mapping: report of the American Academy of Neurology and the American Clinical Neurophysiology Society. Neurology 49, 277–292 10.1212/WNL.49.1.277 9222209

[B69] Oberman L. M. Hubbard E. M. McCleery J. P. Altschuler E. L. Ramachandran V. S. Pineda J. A. (2005). EEG evidence for mirror neuron dysfunction in autism spectrum disorders. Brain Res. Cogn. Brain Res. 24, 190–198 10.1016/j.cogbrainres.2005.01.014 15993757

[B70] Oberman L. M. Ramachandran V. S. Pineda J. A. (2008). Modulation of mu suppression in children with autism spectrum disorders in response to familiar or unfamiliar stimuli: the mirror neuron hypothesis. Neuropsychologia 46, 1558–1565 10.1016/j.neuropsychologia.2008.01.010 18304590

[B71] Orekhova E. V. Stroganova T. A. Nygren G. Tsetlin M. M. Posikera I. N. Gillberg C. (2007). Excess of high frequency electroencephalogram oscillations in boys with autism. Biol. Psychiatry 62, 1022–1029 10.1016/j.biopsych.2006.12.029 17543897

[B72] Orekhova E. V. Stroganova T. A. Prokofyev A. O. Nygren G. Gillberg C. Elam M. (2008). Sensory gating in young children with autism: relation to age, IQ, and EEG gamma oscillations. Neurosci. Lett. 434, 218–223 10.1016/j.neulet.2008.01.066 18313850

[B73] Otnes R. K. Enochson L. (1972). Digital Time Series Analysis. New York: Wiley & Sons

[B74] Pascual-Marqui R. D. Michel C. M. Lehmann D. (1994). Low resolution electromagnetic tomography: a new method for localizing electrical activity in the brain. Int. J. Psychophysiol. 18, 49–65 10.1016/0167-8760(84)90014-X 7876038

[B75] Pineda J. A. Brang D. Hecht E. Edwards L. Carey S. Bacon M. (2008). Positive behavioral and electrophysiological changes following neurofeedback training in children with autism. Res. Autism Spectr. Disord. 2, 557–581 10.1016/j.rasd.2007.12.003

[B76] Pop-Jordanova N. Pop-Jordanov J. (2005). Spectrum-weighted EEG frequency ("brain-rate") as a quantitative indicator of mental arousal. Prilozi 26, 35–42 16400227

[B77] Pop-Jordanova N. Zorcec T. Demerdzieva A. Gucev Z. (2010). QEEG characteristics and spectrum weighted frequency for children diagnosed as autistic spectrum disorder. Nonlinear Biomed. Phys. 4, 4 10.1186/1753-4631-4-4 20920283 PMC2959057

[B78] Postle B. R. D’Esposito M. (2000). Evaluating models of the topographical organization of working memory function in frontal cortex with event-related fMRI. Psychobiology 28, 132–145

[B79] Rihs T. A. Michel C. M. Thut G. (2007). Mechanisms of selective inhibition in visual spatial attention are indexed by alpha-band EEG synchronization. Eur. J. Neurosci. 25, 603–610 10.1111/j.1460-9568.2007.05278.x 17284203

[B80] Rippon G. Brock J. Brown C. Boucher J. (2007). Disordered connectivity in the autistic brain: challenges for the "new psychophysiology." Int. J. Psychophysiol. 63, 164–172 10.1016/j.ijpsycho.2006.03.012 16820239

[B81] Rizzolatti G. Fogassi L. Gallese V. (2001). Neurophysiological mechanisms underlying the understanding and imitation of action. Nat. Rev. Neurosci. 2, 661–670 10.1038/35090060 11533734

[B82] Rojas D. C. Bawn S. D. Benkers T. L. Reite M. L. Rogers S. J. (2002). Smaller left hemisphere planum temporale in adults with autistic disorder. Neurosci. Lett. 328, 237–240 10.1016/S0304-3940(02)00521-9 12147315

[B83] Rojas D. C. Camou S. L. Reite M. L. Rogers S. J. (2005). Planum temporale volume in children and adolescents with autism. J. Autism Dev. Disord. 35, 479–486 10.1007/s10803-005-5038-7 16134033

[B84] Rossignol D. A. Frye R. E. (2012). A review of research trends in physiological abnormalities in autism spectrum disorders: immune dysregulation, inflammation, oxidative stress, mitochondrial dysfunction and environmental toxicant exposures. Mol. Psychiatry 17, 389–401 10.1038/mp.2011.165 22143005 PMC3317062

[B85] Rubenstein J. L. R. Merzenich M. M. (2003). Model of autism: increased ratio of excitation/inhibition in key neural systems. Genes Brain Behav. 2, 255–267 10.1034/j.1601-183X.2003.00037.x 14606691 PMC6748642

[B86] Sauseng P. Klimesch W. Gerloff C. Hummel F. C. (2009). Spontaneous locally restricted EEG alpha activity determines cortical excitability in the motor cortex. Neuropsychologia 47, 284–288 10.1016/j.neuropsychologia.2008.07.021 18722393

[B87] Schipul S. E. Keller T. A. Just M. A. (2011). Inter-regional brain communication and its disturbance in autism. Front. Syst. Neurosci. 5:10 10.3389/fnsys.2011.00010 21390284 PMC3046360

[B88] Sheikhani A. Behnam H. Mohammadi M. R. Noroozian M. Mohammadi M. (2012). Detection of abnormalities for diagnosing of children with autism disorders using of quantitative electroencephalography analysis. J. Med. Syst. 36, 957–963 10.1007/s10916-010-9560-6 20711644

[B89] Sheikhani A. Behnam H. Mohammadi M. R. Noroozian M. (2007). “Analysis of EEG background activity in Autsim disease patients with bispectrum and STFT measure,” in Proceedings of the 11th WSEAS International Conference on COMMUNICATIONS, Agios Nikolaos

[B90] Sheikhani A. Behnam H. Noroozian M. Mohammadi M. R. Mohammadi M. (2009). Abnormalities of quantitative electroencephalography in children with Asperger disorder in various conditions. Res. Autism Spectr. Disord. 3, 538–546 10.1016/j.rasd.2008.11.002

[B91] Sigl J. C. Chamoun N. G. (1994). An introduction to bispectral analysis for the electroencephalogram. J. Clin. Monit. 10, 392–404 10.1007/BF01618421 7836975

[B92] Simonoff E. Pickles A. Charman T. Chandler S. Loucas T. Baird G. (2008). Psychiatric disorders in children with autism spectrum disorders: prevalence, comorbidity, and associated factors in a population-derived sample. J. Am. Acad. Child Adolesc. Psychiatry 47, 921–929 10.1097/CHI.0b013e318179964f 18645422

[B93] Singer W. (1999). Neuronal synchrony: a versatile code for the definition of relations? Neuron 24, 49–65 10.1016/S0896-6273(00)80821-1 10677026

[B94] Steriade M. Gloor P. Llinás R. R. Lopes de Silva F. H. Mesulam M. M. (1990). Report of IFCN Committee on Basic Mechanisms. Basic mechanisms of cerebral rhythmic activities. Electroencephalogr. Clin. Neurophysiol. 76, 481–508 10.1016/0013-4694(90)90001-Z 1701118

[B95] Stroganova T. A. Nygren G. Tsetlin M. M. Posikera I. N. Gillberg C. Elam M. (2007). Abnormal EEG lateralization in boys with autism. Clin. Neurophysiol. 118, 1842–1854 10.1016/j.clinph.2007.05.005 17581774

[B96] Sutton S. K. Burnette C. P. Mundy P. C. Meyer J. Vaughan A. Sanders C. (2005). Resting cortical brain activity and social behavior in higher functioning children with autism. J. Child Psychol. Psychiatry 46, 211–222 10.1111/j.1469-7610.2004.00341.x 15679529

[B97] Thatcher R. W. (1998). Normative EEG databases and EEG biofeedback. J. Neurother. 2, 8–39 10.1300/J184v02n04_02

[B98] Thatcher R. W. North D. M. Neubrander J. Biver C. J. Cutler S. Defina P. (2009). Autism and EEG phase reset: deficient GABA mediated inhibition in thalamo-cortical circuits. Dev. Neuropsychol. 34, 780–800 10.1080/87565640903265178 20183733

[B99] Thatcher R. W. Northa D. Bivera C. (2005). EEG and intelligence: Relations between EEG coherence, EEG phase delay and power. Clin. Neurophysiol. 116, 2129–2141 10.1016/j.clinph.2005.04.026 16043403

[B100] Tierney A. L. Gabard-Durnam L. Vogel-Farley V. Tager-Flusberg H. Nelson C. A. (2012). Developmental trajectories of resting EEG power: an endophenotype of autism spectrum disorder. PLoS ONE 7:e39127 10.1371/journal.pone.0039127 22745707 PMC3380047

[B101] Tong S. Thakor N. V. (2009). Quantitative EEG Analysis Methods and Clinical Applications (Engineering in Medicine & Biology). Boston: Artech House, Inc

[B102] Van Putten M. J. A. M. Peters J. M. Mulder S. M. De Haas J. A. M. Bruijninckx C. M. A. Tavy D. L. J. (2004). A brain symmetry index (BSI) for online EEG monitoring in carotid endarterectomy. Clin. Neurophysiol. 115, 1189–1194 10.1016/j.clinph.2003.12.002 15066544

[B103] Waiter G. D. Williams J. H. G. Murray A. D. Gilchrist A. Perrett D. I. Whiten A. (2004). A voxel-based investigation of brain structure in male adolescents with autistic spectrum disorder. Neuroimage 22, 619–625 10.1016/j.neuroimage.2004.02.029 15193590

[B104] Waiter G. D. Williams J. H. G. Murray A. D. Gilchrist A. Perrett D. I. Whiten A. (2005). Structural white matter deficits in high-functioning individuals with autistic spectrum disorder: a voxel-based investigation. Neuroimage 24, 455–461 10.1016/j.neuroimage.2004.08.049 15627587

[B105] Walter D. O. (1963). Spectral analysis for electroencephalograms: mathematical determination of neurophysiological relationships from records of limited duration. Exp. Neurol. 8, 155–181 10.1016/0014-4886(63)90042-6 20191690

[B106] Welch P. D. (1967). The use of fast Fourier transform for the estimation of power spectra: a method based on time averaging over short, modified periodograms. IEEE Trans. Acoust. AU-15, 70–73 10.1109/TAU.1967.1161901

[B107] Williams J. H. Whiten A. Suddendorf T. Perrett D. I. (2001). Imitation, mirror neurons and autism. Neurosci. Biobehav. Rev. 25, 287–295 10.1016/S0149-7634(01)00014-8 11445135

[B108] Wing L. Gould J. (1979). Severe impairments of social interaction and associated abnormalities in children: epidemiology and classification. J. Autism Dev. Disord. 9, 11–29 10.1007/BF01531288 155684

[B109] Witwer A. N. Lecavalier L. (2008). Examining the validity of autism spectrum disorder subtypes. J. Autism Dev. Disord. 38, 1611–1624 10.1007/s10803-008-0541-2 18327636

[B110] Zhang Y. Chen Y. Bressler S. L. Ding M. (2008). Response preparation and inhibition: the role of the cortical sensorimotor beta rhythm. Neuroscience 156, 238–246 10.1016/j.neuroscience.2008.06.061 18674598 PMC2684699

